# Extending Camera’s Capabilities in Low Light Conditions Based on LIP Enhancement Coupled with CNN Denoising

**DOI:** 10.3390/s21237906

**Published:** 2021-11-27

**Authors:** Maxime Carré, Michel Jourlin

**Affiliations:** 1 NT2I Company, 42000 Saint-Etienne, France; m.carre@nt2i.fr; 2 Hubert Curien Laboratory, Jean Monnet University, 42000 Saint-Etienne, France

**Keywords:** low-light, image enhancement, Logarithmic Image Processing, denoising, CNN, image quality, metrics

## Abstract

Using a sensor in variable lighting conditions, especially very low-light conditions, requires the application of image enhancement followed by denoising to retrieve correct information. The limits of such a process are explored in the present paper, with the objective of preserving the quality of enhanced images. The LIP (Logarithmic Image Processing) framework was initially created to process images acquired in transmission. The compatibility of this framework with the human visual system makes possible its application to images acquired in reflection. Previous works have established the ability of the LIP laws to perform a precise simulation of exposure time variation. Such a simulation permits the enhancement of low-light images, but a denoising step is required, realized by using a CNN (Convolutional Neural Network). A main contribution of the paper consists of using rigorous tools (metrics) to estimate the enhancement reliability in terms of noise reduction, visual image quality, and color preservation. Thanks to these tools, it has been established that the standard exposure time can be significantly reduced, which considerably enlarges the use of a given sensor. Moreover, the contribution of the LIP enhancement and denoising step are evaluated separately.

## 1. Introduction

In the present paper, our main goal is to study the case of extremely low-light images acquired with a very short exposure time, up to fifty times smaller than a standard one. We already knew that the LIP tools permit precisely simulating a target exposure time, thus performing an accurate brightness correction of low-light images. Many other enhancement solutions are proposed in the literature, and most of them take into account a specific knowledge of the studied domain, for example, kind of noise, sought information, etc. Let us cite, among many examples [1] (mammographic images), [2] (echo imaging of the abdomen), and [3] (image quality for mobile devices). Some recent papers propose general solutions, most of the time based on Neural Networks [4,5]. This amount of papers is due to the various domains concerned, night photography, night vision, astronomy, and the reduction of X-rays doses in medical imaging, among others.

The paper is organized as follows. Section 2 is dedicated to LIP tools recalls. Section 3 and Section 4 present their application to image enhancement and exposure time simulation. In Section 5, the CNN denoising approach we have selected is described. In Section 6, we test three objective parameters (PSNR, SSIM, and DeltaE) on the same image acquired under a large range of exposure times, and we show that classic exposure times (around 50 ms) can be divided by 5 without a significant loss of quality.

## 2. LIP Bases

The Logarithmic Image Processing (LIP) model was first defined by Jourlin and Pinoli [6,7,8] for grey-level images. For interested readers, a book has been entirely dedicated to the model [9]. The LIP framework is founded on strong mathematical and physical justifications. In fact, it was initially dedicated to images acquired in transmission (when the observed object is located between the source and the sensor). Then, Brailean [10] established the consistency of the LIP Model with the Human Visual System, which enlarges its application field to images acquired in reflection; in fact, LIP operators allow analyzing and interpreting such images like a human eye would. This property is a consequence of the Weber–Fechner laws (see for example [11]).

Due to the *Transmittance Law*, two operations have been proposed on the space *I*(*D*, [0, *M*]) of grey-level images defined on the same spatial domain *D*, with values in the greyscale [0, *M*]:

The *addition* of two images *f* and *g* according to:

(1) $$f⨹g=f+g-\frac{f\cdot g}{M}$$

The *scalar multiplication* of an image *f* by a real number *λ* according to:

(2) $$\lambda ⨻f=M-M{\left(1-\frac{f}{M}\right)}^{\lambda \;\;\;\;}$$

**Figure 1:**
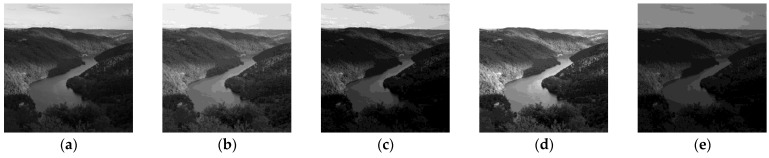
Brightening/darkening an image thanks to LIP laws. (**a**) Original image *f*; (**b**) Image 0.75 ⨻ *f*; (**c**) Image 1.25 ⨻ *f*; (**d**) Image *f* ⨺ 100; (**e**) Image *f* ⨹ 100.

**Remark** **1.** *In the situation of transmitted light, which means that the observed object is half-transparent, Formula (2) is easily interpretable. It consists of multiplying the object thickness by λ. Thus, it becomes obvious that λ *⨻* f is darker than f for λ *≥* 1 and brighter than f if λ ≤ 1 (cf. Figure 1a).*

**Remark** **2.** *If λ takes negative values, λ *⨻* f is no more an image but a function, element of the set F(D, [−∞, M]). It has been established (see [7] for example) that the space of functions [F(D, [−∞, M]), *⨹*, *⨻*] equipped with the laws *⨹* and *⨻* becomes a Real Vector Space. The space of images I(D, [0, M]) appears as the Positive Cone of this Vector Space. For the understanding of this paper, this remark is not essential, but it shows that we can use with confidence the numerous tools that mathematicians have defined in Vector Spaces, like interpolation, scalar product, and norms.*

**Remark** **3.** *In the Vector Space [F(D, [−∞, M]), *⨹*, *⨻*], it is possible to define the opposite of an image f, according to:*

(3) $$⨺f=\frac{-f}{1-\frac{f}{M}}$$

*which satisfies:*

(4) f⨹(⨺f)=0

*and the subtraction between two images:*

(5) $$f⨺g=\frac{f-g}{1-\frac{g}{M}}$$

*which is an image if f(x) *≥* g(x) for every x of D. If not, f *⨺* g is a function of F(D, [−∞, M]) which can take negative values.*

**Remark** **4.** *Inside the LIP framework, the greyscale is inverted compared to the classical convention. In fact, 0 represents the bright extremity corresponding to the source intensity (when the observed object is fully transparent). The reason is that in such conditions, 0 appears as the neutral element of the addition *⨹*. Moreover, negative values of the interval ([−∞, M]) must be interpreted as light intensifiers, as shown by Formula (4). Adding negative values to an image f can result in null grey levels, i.e., transparent ones.*

## 3. Low-Light Images Enhancement

Once the base of the LIP framework was established, many applications were developed in image processing. From the beginning, it appeared that brightness modification, and especially image enhancement, would be a central use of LIP operators (cf. [12]). In fact, each of the LIP basic laws can brighten a dark image: cf. *Remark 1* for the scalar multiplicative law. Concerning the addition law, it is clear that if an image presents the same grey level *C* at each point, the addition of *C* to a given image *f* will darken *f* while the subtraction of *C* will brighten *f* (cf. Figure 1b). Note that the multiplicative law produces a logarithmic enhancement while the addition law produces a linear one.

Among many papers dedicated to LIP enhancement, we can cite the works of two teams headed by Cahill and Deng [13,14,15] and Agaian and Panetta [16,17]. Concerning LIP enhancement of color images, we can refer to [18,19,20,21] and to the LIP book [9].

## 4. Exposure Time Simulation

**Figure 2:**
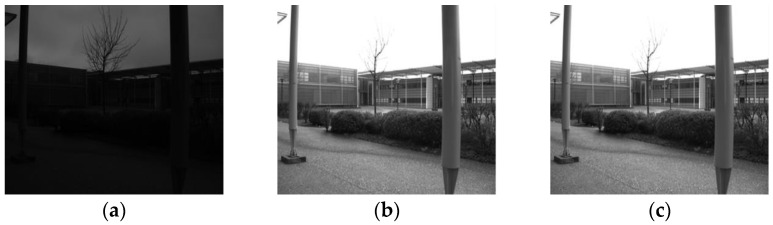
Ability of LIP subtraction of a constant to simulate exposure time changing. (**a**) Image *f*, “Laboratory” acquired with exposure time = 10 ms; (**b**) Image *g*, “Laboratory” acquired with exposure time = 100 ms; (**c**) Starting from *f*, simulation of an exposure time of 100 ms.

**Figure 3:**
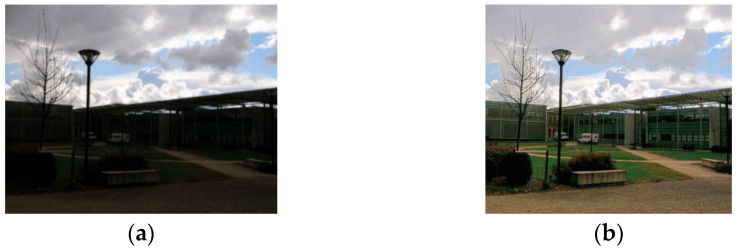
LIP tone mapping algorithm using LIP subtractions to enhance a color low-light image. (**a**) Original image; (**b**) LIP Tone Mapping applied on (**a**).

In the majority of the aforementioned methods, the enhancement step was not driven by a rigorous goal but aimed at making low-light images visually interpretable. To improve such an approach, Carré established that it was possible to perfectly simulate variations of exposure time by performing LIP addition/subtraction of a constant [22,23]. This applies to grey level images (cf. Figure 2) as well as color images by working on the associated luminance image computed as the mean of the 3 channels Red, Green, and Blue, and the LIP addition/subtraction being performed on each of the 3 channels (cf. Figure 3, an example of a LIP tone mapping algorithm using LIP subtraction on color images [18]).

**Figure 4:**
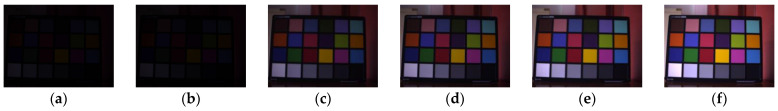
Color chart acquired at various exposure times. (**a**) image *f*_3_ at 3 ms; (**b**) image *f*_6_ at 6 ms; (**c**) image *f*_18_ at 18 ms; (**d**) image *f*_24_ at 24 ms; (**e**) image *f*_33_ at 33 ms; (**f**) image *f*_45_ at 45 ms.

These brightness corrections can be automated in many ways. We propose here an algorithm searching the “optimal” constant to subtract (in the LIP sense) from the image that maximizes the standard deviation of its histogram. We consider a Color Chart acquired at exposure times varying from 3 to 45 ms (Figure 4). The range of exposure times is deliberately large to test the efficiency of simulation images.

Now, we associate to a given color image *f* its *Global Histogram* denoted *GH_f_* which consists of cumulating the histograms of the three channels R, G, B. It is then possible to associate to *f* an enhanced image *f* ⨺ *C*_1_. The constant *C*_1_ is computed to maximize the standard deviation *σ*[*GH_f_* ⨺ *C*] for all the possible values of *C* ∊ [0, *M*]. In other words, *C*_1_ satisfies:

(6) $$\sigma \left[GH_{f}⨺C_{1}\right]=Sup_{C\in [0,M[}\left\{\sigma \left[GH_{f}⨺C\right]\right\}$$

**Figure 5:**
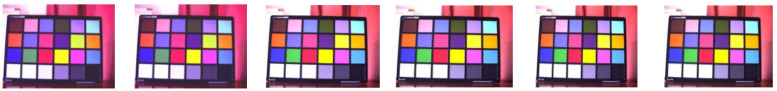
Enhanced images associated to *f*_3_, *f*_6_, *f*_18_, *f*_24_, *f*_33_, *f*_45_, from left to right.

Let us remark that, contrary to a method of histogram equalization, for example, the resulting enhanced image is visually independent of the initial exposure time (Figure 5).

**Figure 6:**
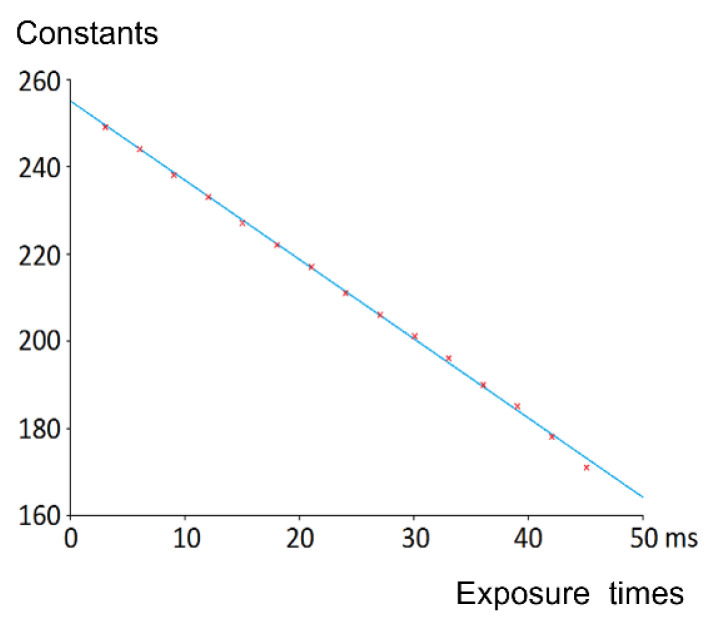
Proof of the linear relation between the exposure time and the constant grey level subtracted from the acquired image to get the enhanced one.

More precisely, we want to study how these constants vary according to the corresponding exposure times, and we expect that the relationship is a linear one due to the expression of the LIP subtraction. To strengthen this hypothesis, more acquisitions of the color chart have been realized, namely 15 acquisitions with exposure times from 3 ms to 45 ms (3 ms, 6 ms, 9 ms, 12 ms, 15 ms, 18 ms, 21 ms, 24 ms, 27 ms, 30 ms, 33 ms, 36 ms, 39 ms, 42 ms, and 45 ms). Figure 6 confirms the linearity of the relation.

## 5. CNN Denoising

The enhancement of an image requires a denoising step, particularly when it is realized on a very low-light image. There are many denoising approaches. Some are very simple and consist, for example, of applying a *Median filter* which attenuates the salt and pepper noise while preserving the boundaries. Other methods aim at modeling a specific noise. A classic overview of the subject is given in [24]. Recently, Tian et al. published a survey dedicated to *Deep learning image denoising* [25].

Let us now present examples of such methods. Based on a U-Net architecture [26], Lehtinen proposes a novel approach called “Noise2Noise” that aims at denoising images by only looking at noisy samples [27]. While many Deep Learning denoising methods learn pairs of noisy (input)/clean images (output), this solution needs only pairs of noisy images acquired under the same conditions such that only the noise changes; the network is trained with a noisy image in input and the other one in output. Once the network is trained, a noisy image in the input results in a clean output. This approach facilitates the creation of the dataset required to learn and correct the noise of a specific sensor. This is the reason why we have selected the Lehtinen method.

In our case, we aim to stabilize the brightness of images acquired in various conditions. For example, in low light conditions, high dynamic scenes (LIP correction and CNN denoising have been applied to tone mapping problematics in [28]), etc. Since our stabilization enhances the signal and the noise level at the same time, the intensity of the noise varies depending on the acquisition conditions. Practically, we train the network on images acquired in different conditions to learn different levels of noise. Each image pair of our training dataset is represented by two iterations of the same scene on which LIP stabilization is applied.

In addition to increased noise, enhancing an image dynamic generates a quantization effect. The histogram of a dark image is initially compressed, and the greyscale is sampled on a number of bits (8 bits in our case). Thus, our transformation leads to an expanded but quantized histogram. On very low-light enhanced images, this quantization can be visible. It has been shown [28] that this denoising technique corrects this effect. That is why the denoising step must be applied after the enhancement step.

**Figure 7:**
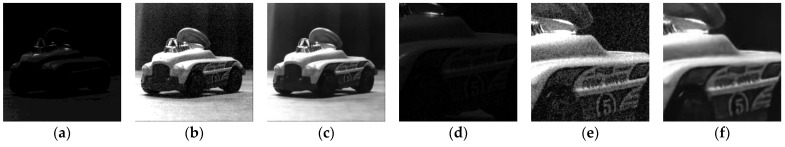
Grey level image enhancement. (**a**) image acquired at low exposure; (**b**) LIP enhancement of (**a**); (**c**) CNN denoising applied on (**b**); (**d**) zoom on (**a**); (**e**) zoom on (**b**); (**f**) zoom on (**c**).

**Figure 8:**
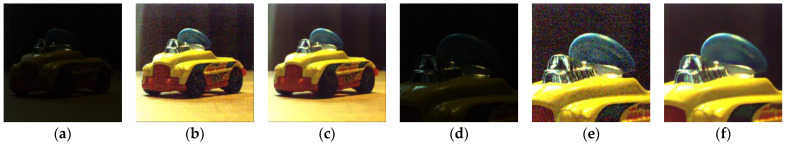
Color image enhancement. (**a**) image acquired at low exposure; (**b**) LIP enhancement of (**a**); (**c**) CNN denoising applied on (**b**); (**d**) zoom on (**a**); (**e**) zoom on (**b**); (**f**) zoom on (**c**).

Some results are presented for a grey level image (Figure 7) and for a color image (Figure 8). In this last case, the LIP enhancement is applied to the associated luminance plane. The visual results are promising but must be confirmed by objective parameters. This is the object of the following section.

## 6. Evaluation of Images Quality

In this section, we propose a comparison between enhanced noisy images (LIP stabilization + CNN denoising) and clean targets (images acquired under normal conditions). Our evaluations depend on three measures: PSNR (Peak Signal to Noise Ratio), which is commonly used in the literature and efficient to estimate the noise level, but not representative of human perception; SSIM (Structural Similarity Index), considered a metric that aims at analyzing structural changes between two images to get a measure closer to human consideration; and the DeltaE measure to analyze the color accuracy of the proposed solution.

### 6.1. Training

Using Basler acA 1300–30gc camera (Basler AG, 22926 Ahrensburg, Germany) with fixed aperture f/4, multiple image pairs of various scenes were acquired. Different exposure times were used to get many brightness variations (from correctly balanced images to very low light images). An image pair is constituted of two images acquired in the same conditions with the same exposure time; only the image noise differs between these two successive frames. All images are enhanced by LIP subtraction to reach a grey level average of 100. The dataset comprises 564 acquisitions cropped into 3384 images with 512 × 512 resolution (1692 pairs).

We used the network architecture (U-Network) and the training parameters described in the “Noise2Noise” paper [27]: L2 loss, batch size 4, learning rate 0.001, Adam optimizer with *β*_1_ = 0.9 and *β*_2_ = 0.99.

### 6.2. Experiment

**Figure 9:**
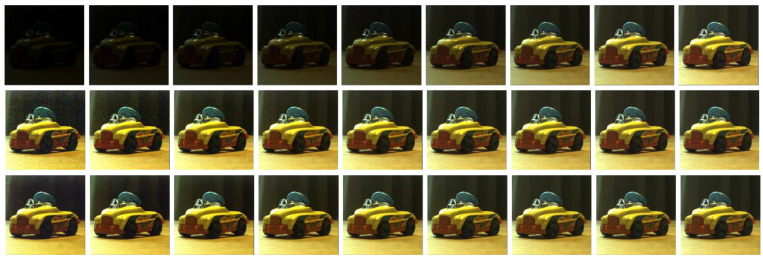
Acquisitions from 1.11 ms to 50 ms. Top: Original images; Middle; LIP stabilization; Bottom: LIP + CNN denoising.

Using the same acquisition conditions as the training step, a scene was acquired under 9 different exposure times from 1 ms to 50 ms. Each image was enhanced by LIP subtraction to reach an average luminance equal to that of the image acquired at 50 ms; this last one is considered as the reference image. Then, the CNN denoising step was applied on each LIP enhanced image (Figure 9).

For each processing step (raw image, LIP enhanced image, and LIP + CNN denoising), PSNR, SSIM, and Delta E were measured between each image and the clean target. The clean target corresponds to the image acquired with the higher exposure time (50 ms) on which has been applied the denoising operation (LIP enhancement is not required on this image since its average is the reference). Despite its good quality, a subtle noise exists on this image, which is cleared by denoising.

### 6.3. Results

**Figure 10:**
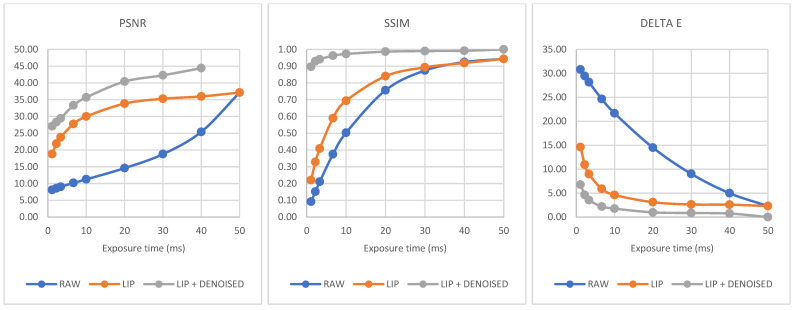
Evolution of PSNR, SSIM, and Delta E according to exposure time for each processing step (raw image, LIP enhancement, LIP + CNN denoising. For PSNR, LIP + denoised value is not represented for the clean target (50 ms) because it reaches infinity.

The measures are presented in Table 1. For each processing step, Figure 10 shows the evolution of the different measures according to exposure times.

### 6.4. Discussion

First, let us explain the choice of the three considered parameters. It is known that PSNR is not efficient for evaluating the quality of an image in the absolute because it does not consider human visual perception. Nevertheless, PSNR is a classical way to compare different restorations of the same image. In complement to PSNR, we selected SSIM, which was first defined to estimate the performance of image compression methods in terms of visual quality. Finally, we have retained Delta E, a measure defined by the International Commission on Illumination, that quantifies the difference between a displayed color and the original one. A low Delta E value corresponds to good accuracy, while a high one reveals a significant mismatch.

Figure 10 puts in evidence the separate contributions of LIP enhancement and CNN denoising.

For PSNR, the LIP enhancement produces values higher than 30 for exposure times greater than 10 ms. This value of 30 is reached for exposure times greater than 3.33 ms when we combine enhancement and denoising. Concerning the SSIM, the improvement of image quality is clearly significant. We remark the weakness of the LIP contribution for very short exposure times, despite the consistency of the LIP Model with human vision. In fact, with the noise and the signal enhanced together, the denoising step becomes essential. The combination of LIP enhancement and CNN denoising produces SSIM values greater than 0.90 for all the studied exposure times. Finally, the curve representing the Delta E evolution shows the importance of the LIP contribution, producing small values (less than 9) of this metric for exposure times smaller than 3.33 ms. From these results, we observe that the couple LIP/denoising appears highly effective for SSIM and Delta E.

It would be important to test our enhancement experiments on a public low-light image database. Unfortunately, our approach requires training on pairs of noisy images of the same scene acquired under the same conditions in order to apply the “Noise2Noise” method. Nevertheless, our images can be found at [29].

A situation for which the denoising step is particularly useful deals with motion acquisition, presented in the next section.

## 7. Application to Motion Acquisition

**Figure 11:**
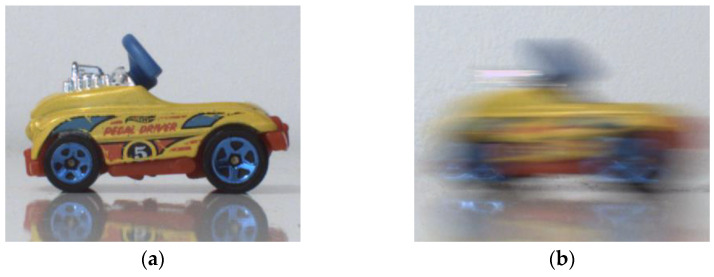
Example of motion acquisition with a standard exposure time (here 50 ms). (**a**) Acquisition of a motionless car; (**b**) Acquisition of a moving car.

**Figure 12:**
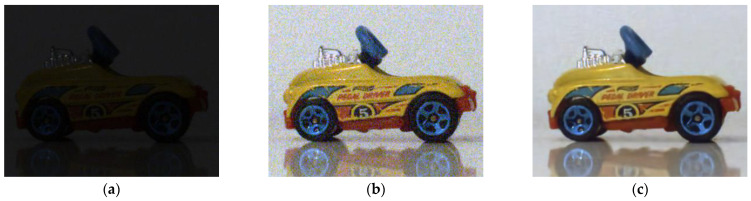
Short exposure acquisition (2 ms) on a moving object and application of LIP enhancement + denoising. (**a**) Motion acquisition with a short exposure time; (**b**) Noisy image after LIP enhancement; (**c**) Image after enhancement and denoising.

**Figure 13:**
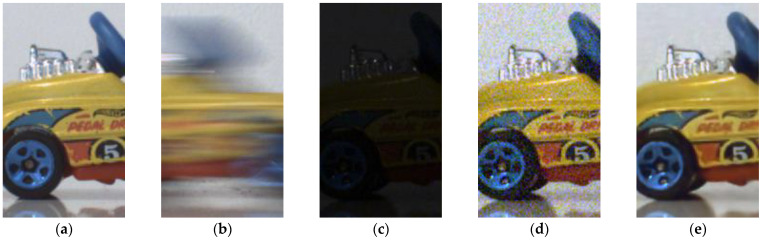
Zoom on Figure 11 and Figure 12. (**a**) Acquisition of a motionless car at 50 ms; (**b**) Acquisition of a moving car at 50 ms; (**c**) Motion acquisition with a short exposure time (2 ms); (**d**) Noisy image after LIP enhancement; (**e**) Image after enhancement and denoising.

To avoid motion blur (Figure 11b), acquiring the image of a moving object necessitates a short exposure time, which results in a very dark image (Figure 12a). This situation corresponds perfectly to what has been described previously: the dark image can be enhanced by means of LIP subtraction of a constant to simulate an exposure time of 50 ms. The brightness of the resulting image is corrected (Figure 12b), but the resulting image is noisy. After denoising, we obtain a result visually similar to the image acquired without movement at a standard exposure time (Figure 12c). For more detailed images, zoomed versions are proposed (Figure 13).

## 8. Conclusions

Images acquired under very short exposure times and/or degraded lighting conditions need a preprocessing step to get a reliable stabilization of their brightness. Previous works [22,23] have demonstrated the effectiveness of LIP tools to perform such a brightness correction, even in extreme conditions of very low-light images. Compared to classical enhancement methods, the LIP approach presents two major advantages: it permits precise modeling of variable exposure times and has been demonstrated to be consistent with human vision [10]. Nevertheless, it does not avoid the recurrent drawback of enhancement algorithms: the noise and the signal are enhanced together, requiring an adapted denoising phase. In the present paper, a CNN has been trained to learn various noise levels. In addition to LIP enhancement, CNN denoising produces images of excellent visual quality. To make this evaluation objective, three factors were selected: the classical PSNR, which is well adapted to denoising measurement; the classical SSIM parameter, which takes into account the human visual system; and finally, the Delta E parameter, which estimates the quality of color preservation. These measures have shown that the proposed solution effectively preserves the colorimetry and the structure of images acquired under significant lighting variations. Moreover, this method can be easily adapted to another camera and is compatible with real-time situations.

An application to motion acquisition has been described, which comes perfectly within the target of the proposed method because it requires a very short exposure time to get clear images.

A more general problem consists of taking into account speed, luminance, and aperture at the same time. A solution is proposed by the APEX (Additive System of Photo-graphic EXposure) equation [30]. Thus, it would be interesting to investigate how our approach could be interpreted in this wider context. The main problem to overcome is the depth of field modification generated by aperture changing.

## Figures and Tables

**Table 1:** PSNR, SSIM, and Delta E values according to images exposures and processing step (raw, LIP enhancement, and LIP + CNN denoising).

Expo (ms)	PSNR			SSIM			DeltaE		
	Raw	LIP	LIP + Denois.	Raw	LIP	LIP + Denois.	Raw	LIP	LIP + Denois.
50	37.13	37.13	inf	0.94	0.94	1.00	2.30	2.30	0.00
40	25.40	35.97	44.41	0.92	0.92	0.99	4.98	2.58	0.74
30	18.76	35.29	42.28	0.87	0.89	0.99	9.02	2.63	0.85
20	14.60	33.83	40.41	0.76	0.84	0.99	14.49	3.10	0.98
10	11.25	30.01	35.69	0.50	0.69	0.97	21.66	4.60	1.76
6.66	10.18	27.75	33.30	0.37	0.59	0.96	24.64	5.89	2.19
3.33	9.02	23.79	29.42	0.21	0.41	0.94	28.15	8.97	3.53
2.22	8.59	21.85	28.31	0.15	0.33	0.93	29.45	10.94	4.63
1.11	8.10	18.79	27.07	0.09	0.22	0.90	30.81	14.61	6.77

## Data Availability

The test data presented in this study are available in [29]. Additional materials can be requested from the authors.
